# Public Health and Economic Consequences of Methyl Mercury Toxicity to the Developing Brain

**DOI:** 10.1289/ehp.7743

**Published:** 2005-02-28

**Authors:** Leonardo Trasande, Philip J. Landrigan, Clyde Schechter

**Affiliations:** ^1^Center for Children’s Health and the Environment, Department of Community and Preventive Medicine, and; ^2^Department of Pediatrics, Mount Sinai School of Medicine, New York, New York, USA;; ^3^Division of General Pediatrics, Children’s Hospital, Boston, Massachusetts, USA;; ^4^Department of Pediatrics, Harvard Medical School, Boston, Massachusetts, USA;; ^5^Department of Family Medicine, Albert Einstein College of Medicine, Bronx, New York, USA

**Keywords:** children’s health, cognitive development, cord blood, electrical generation facilities, environmentally attributable fraction, fetal exposure, lost economic productivity, mercury, methyl mercury, power plants

## Abstract

Methyl mercury is a developmental neurotoxicant. Exposure results principally from consumption by pregnant women of seafood contaminated by mercury from anthropogenic (70%) and natural (30%) sources. Throughout the 1990s, the U.S. Environmental Protection Agency (EPA) made steady progress in reducing mercury emissions from anthropogenic sources, especially from power plants, which account for 41% of anthropogenic emissions. However, the U.S. EPA recently proposed to slow this progress, citing high costs of pollution abatement. To put into perspective the costs of controlling emissions from American power plants, we have estimated the economic costs of methyl mercury toxicity attributable to mercury from these plants. We used an environmentally attributable fraction model and limited our analysis to the neurodevelopmental impacts—specifically loss of intelligence. Using national blood mercury prevalence data from the Centers for Disease Control and Prevention, we found that between 316,588 and 637,233 children each year have cord blood mercury levels > 5.8 μg/L, a level associated with loss of IQ. The resulting loss of intelligence causes diminished economic productivity that persists over the entire lifetime of these children. This lost productivity is the major cost of methyl mercury toxicity, and it amounts to $8.7 billion annually (range, $2.2–43.8 billion; all costs are in 2000 US$). Of this total, $1.3 billion (range, $0.1–6.5 billion) each year is attributable to mercury emissions from American power plants. This significant toll threatens the economic health and security of the United States and should be considered in the debate on mercury pollution controls.

Mercury is a ubiquitous environmental toxicant (Goldman et al. 2001). It exists in three forms, each of which possesses different bioavailability and toxicity: the metallic element, inorganic salts, and organic compounds (methyl mercury, ethyl mercury, and phenyl mercury) (Franzblau 1994). Although volcanoes and other natural sources release some elemental mercury to the environment, anthropogenic emissions from coal-fired electric power generation facilities, chloralkali production, waste incineration, and other industrial activities now account for approximately 70% of the 5,500 metric tons of mercury that are released into the earth’s atmosphere each year [United Nations Environmental Programme (UNEP) 2002]. Elemental mercury is readily aerosolized because of its low boiling point, and once airborne it can travel long distances to eventually deposit into soil and water. In the sediments of rivers, lakes, and the ocean, metallic mercury is transformed within microorganisms into methyl mercury (Guimaraes et al. 2000). This methyl mercury biomagnifies in the marine food chain to reach very high concentrations in predatory fish such as swordfish, tuna, king mackerel, and shark (Dietz et al. 2000; Gilmour and Riedel 2000; Mason et al. 1995; Neumann and Ward 1999). Consumption of contaminated fish is the major route of human exposure to methyl mercury.

The toxicity of methyl mercury to the developing brain was first recognized in the 1950s in Minamata, Japan, where consumption of fish with high concentrations of methyl mercury by pregnant women resulted in at least 30 cases of cerebral palsy in children; exposed women were affected minimally if at all (Harada 1968). A similar episode followed in 1972 in Iraq when the use of a methyl mercury fungicide led to poisoning in thousands of people (Bakir et al. 1973); again, infants and children were most profoundly affected (Amin-Zaki et al. 1974, 1979). The vulnerability of the developing brain to methyl mercury reflects the ability of lipophilic methyl mercury to cross the placenta and concentrate in the central nervous system (Campbell et al. 1992). Moreover, the blood–brain barrier is not fully developed until after the first year of life, and methyl mercury can cross this incomplete barrier (Rodier 1995).

Three recent, large-scale prospective epidemiologic studies have examined children who experienced methyl mercury exposures *in utero* at concentrations relevant to current U.S. exposure levels. The first of these studies, a cohort in New Zealand, found a 3-point decrement in the Wechsler Intelligence Scale-Revised (WISC-R) full-scale IQ among children born to women with maternal hair mercury concentrations > 6 μg/g (Kjellstrom et al. 1986, 1989). A second study in the Seychelles Islands in the Indian Ocean found only one adverse association with maternal hair mercury concentration among 48 neurodevelopmental end points examined (prolonged time to complete a grooved pegboard test with the nonpreferred hand) (Myers et al. 2003). However, the grooved pegboard test was one of the few neurobehavioral instruments in the Seychelles study not subject to the vagaries of translation that can degrade the validity of culture-bound tests of higher cognitive function when they are applied in developing nations (Landrigan and Goldman 2003). A third prospective study in the Faroe Islands, a component of Denmark inhabited by a Scandinavian population in the North Atlantic, has followed a cohort of children for 14 years and collected data on 17 neurodevelopmental end points, as well as on the impact of methyl mercury on cardiovascular function. The Faroes researchers found significant dose-related, adverse associations between prenatal mercury exposure and performance on a wide range of memory, attention, language, and visual-spatial perception tests (Grandjean et al. 1997). The significance of these associations remained evident when blood levels of polychlorinated biphenyls, which are known developmental neurotoxicants (Jacobson and Jacobson 1996), were included in the analysis (Budtz-Jorgensen et al. 2002; Steuerwald et al. 2000). Methyl mercury exposure was also associated with decreased sympathetic- and parasympathetic-mediated modulation of heart rate variability (Grandjean et al. 2004) and with persistent delays in peaks I–III brainstem evoked potentials (Murata et al. 2004).

An assessment of these three prospective studies by the National Academy of Sciences (NAS) (National Research Council 2000) concluded that there is strong evidence for the fetal neurotoxicity of methyl mercury, even at low concentrations of exposure. Moreover, the NAS opined that the most credible of the three prospective epidemiologic studies was the Faroe Islands investigation. In recommending a procedure for setting a reference dose for a methyl mercury standard, the NAS chose to use a linear model to represent the relationship between mercury exposure and neurodevelopmental outcomes, and based this model on the Faroe Islands data. The NAS found that the cord blood methyl mercury concentration was the most sensitive biomarker of exposure *in utero* and correlated best with neurobehavioral outcomes. The NAS was not deterred by the apparently negative findings of the Seychelles Islands study, which it noted was based on a smaller cohort than the Faroe Islands investigation and had only 50% statistical power to detect the effects observed in the Faroes (National Research Council 2000).

Since January 2003, the issue of early life exposure to methyl mercury has become the topic of intense debate after the U.S. Environmental Protection Agency (EPA) announced a proposal to reverse strict controls on emissions of mercury from coal-fired power plants. This proposed “Clear Skies Act” would slow recent progress in controlling mercury emission rates from electric generation facilities and would allow these releases to remain as high as 26 tons/year through 2010 (U.S. EPA 2004a). By contrast, existing protections under the Clean Air Act will limit mercury emissions from coal-fired power plants to 5 tons/year by 2008 (U.S. EPA 2004b). The U.S. EPA’s technical analyses in support of “Clear Skies” failed to incorporate or quantify consideration of the health impacts resulting from increased mercury emissions (U.S. EPA 2004c). After legislative momentum for this proposal faded, the U.S. EPA proposed an almost identical Utility Mercury Reductions Rule, which again failed to examine impacts on health. The U.S. EPA issued a final rule on 15 March 2005 (U.S. EPA 2005).

To assess the costs that may result from exposure of the developing brain to methyl mercury, we estimated the economic impact of anthropogenic methyl mercury exposure in the 2000 U.S. birth cohort. We calculated the fraction of this cost that could be attributed to mercury emitted by American electric power generation facilities.

## Materials and Methods

### Environmentally attributable fraction model.

To assess the disease burden and the costs due to methyl mercury exposure, we used an environmentally attributable fraction (EAF) model. The EAF approach was developed by the Institute of Medicine (IOM) to assess the “fractional contribution” of the environment to causation of illness in the United States (IOM 1981), and it has been used to assess the costs of environmental and occupational disease (Fahs et al. 1989; Leigh et al. 1997). It was used recently to estimate the environmentally attributable costs of lead poisoning, asthma, pediatric cancer, and neurodevelopmental disabilities in American children (Landrigan et al. 2002). The EAF is defined by Smith et al. (1999) as “the percentage of a particular disease category that would be eliminated if environmental risk factors were reduced to their lowest feasible concentrations.” The EAF is a composite value and is the product of the prevalence of a risk factor multiplied by the relative risk of disease associated with that risk factor. Its calculation is useful in developing strategies for resource allocation and prioritization in public health. The general model developed by the IOM and used in the present analysis is the following:


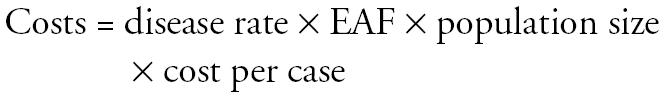


“Cost per case” refers to discounted lifetime expenditures attributable to a particular disease, including direct costs of health care, costs of rehabilitation, and lost productivity. “Disease rate” and “population size” refer, respectively, to the incidence or prevalence of a disease and the size of the population at risk.

In applying the EAF model, we first reviewed the adverse effects of methyl mercury exposure. We then estimated the costs of those effects and subsequently applied a further fraction to parse out the cost of anthropogenic methyl mercury exposure resulting from emissions of American electrical generation facilities.

### Toxic effects of methyl mercury exposure.

The NAS found neurodevelopmental effects in the children of women who had consumed fish and seafood during pregnancy to be the most important and best-studied end point for methyl mercury toxicity. Although the NAS identified other potentially significant toxicities resulting from methyl mercury exposure, such as nephrotoxicity and carcinogenicity, those effects were less well characterized (National Research Council 2000). We therefore limited our analysis to the neurodevelopmental impact of methyl mercury toxicity.

There is no evidence to date validating the existence of a threshold blood mercury concentration below which adverse effects on cognition are not seen. The U.S. EPA has, however, set a benchmark dose level (BMDL) for cord blood mercury dose concentration of 58 μg/L. This level that corresponds to the lower limit of the 95% confidence interval for the concentration at which there is a doubling in the Faroes study in the prevalence of test scores (5–10%) in the clinically subnormal range for the Boston Naming Test (Rice et al. 2003). It is important to note that this is not a concentration below which no observed adverse effects were found. The Faroes and New Zealand cohorts both support the conclusion that developmental effects become apparent at levels of approximately 1 ppm mercury in hair, or 5.8 μg/L in cord blood (Grandjean et al. 1997; Kjellstrom et al. 1986, 1989). The Faroes study also found that effects on delayed brainstem auditory responses occurred at much lower exposure concentrations (Murata et al. 2004). In its report, the NAS concluded that the likelihood of subnormal scores on neurodevelopmental tests after *in utero* exposure to methyl mercury increased as cord blood concentrations increased from levels as low as 5 μg/L to the BMDL of 58 μg/L (National Research Council 2000). In light of those findings, we decided in this analysis to apply a no adverse effect level of 5.8 μg/L, the lowest level at which adverse neurodevelopmental effects were demonstrated in the cohort studies.

Recent data suggest that the cord blood mercury concentration may on average be 70% higher than the maternal blood mercury concentration (Stern and Smith 2003), and a recent analysis suggests that a modification of the U.S. EPA reference dose for methyl mercury be made to reflect a cord blood:maternal blood ratio that is > 1 (Stern 2005). If the developmental effects of mercury exposure do, in fact, begin at 5.8 μg/L in cord blood, as suggested by the Faroes (Grandjean et al. 1997) and New Zealand (Kjellstrom et al. 1986, 1989) data and by the NAS report (National Research Council 2000), then effects would occur in children born to women of child-bearing age with blood mercury concentrations ≥3.41 (ratio, 5.8:1.7) μg/L. National population data from the 1999–2000 National Health and Nutrition Examination Survey (NHANES) found that 15.7% of American women of childbearing age have total blood mercury concentrations ≥3.5 μg/L (Mahaffey et al. 2004).

To compute IQ decrements in infants that have resulted from these elevated maternal mercury exposures, we used published data on percentages of women of childbearing age with mercury concentrations ≥3.5, 4.84, 5.8, 7.13, and 15.0 μg/L. We assumed conservatively that all mercury concentrations within each of the segments of the distribution were at the lower bound of the range. We assumed that the probability of giving birth to a child did not correlate with mercury level in a woman of childbearing age. In our base case analysis, we calculated economic costs assuming that children born to women with mercury concentrations 3.5–4.84 μg/L suffer no loss in cognition, and that successive portions of the birth cohort experience loss of cognition associated with cord blood levels of 8.2, 9.9, 12.1, and 25.5 μg/L, respectively.

Recently, the Faroes researchers reviewed their cohort data and found fetal blood mercury concentrations to be only 30% higher than maternal blood concentrations (Budtz-Jorgensen et al. 2004). In light of these findings and to avoid overestimation of the magnitude of impacts, we chose not to include children born to mothers with blood mercury concentrations between 3.5 and 4.84 μg/L in our base case analysis.

To assess the impact on our findings of a range of various possible ratios between maternal and cord blood mercury concentrations, we conducted a sensitivity analysis. In this analysis, we set as a lower bound for our estimate the costs to children with estimated cord blood concentrations ≥5.8 μg/L (assuming a cord:maternal blood ratio of 1) and assumed no IQ impact < 4.84 μg/L (assuming a cord:maternal blood ratio of 1.19). This estimate assumed no loss of cognition to children born to women with mercury concentration < 5.8 μg/L and assumed that subsequent portions of the birth cohort experienced cord blood mercury concentrations of 5.8, 7.13, and 15 μg/L, respectively. To estimate economic costs in this scenario, we calculated no costs for children with blood mercury concentrations < 4.84 μg/L. We calculated costs resulting from an incremental increase in blood mercury concentration from 4.84 to 5.8 μg/L in the percentage of the population with blood mercury levels between 5.8 and 7.13 μg/L, and added those costs to the costs resulting from increases from 4.84 to 7.13 μg/L and 4.84 to 15 μg/L in the percentages of the population with concentrations between 7.13 and 15 μg/L and > 15 μg/L, respectively. The result of this calculation is expressed in our analysis as a lower bound for the true economic cost of methyl mercury toxicity to the developing brain.

### Impact of methyl mercury exposure on IQ.

The Faroes study found that a doubling of mercury concentration was associated with adverse impacts on neurodevelopmental tests ranging from 5.69–15.93% of a standard deviation (Grandjean et al. 1999). Assuming that IQ is normally distributed with a standard deviation of 15 points, a doubling of mercury concentration would be associated with a decrement ranging from 0.85 to 2.4 IQ points. The Faroes researchers used a structural equation analysis to produce estimates of impact of methyl mercury on verbal and motor function at 7 years of age and found an association between a doubling of blood mercury and loss of 9.74% of a standard deviation on motor function and of 10.45% of a standard deviation on verbal function (Budtz-Jorgensen et al. 2002). This analysis suggests that a doubling in mercury concentration produces a decrement of approximately 10% of a standard deviation, or 1.5 IQ points. In the New Zealand study (Kjellstrom et al. 1986, 1989), the average WISC-R full-scale IQ for the study population (*n* = 237) was 93. In the group with maternal hair mercury > 6 μg/g (~ 4-fold higher than in the study population, *n* = 61), the average was 90 (Kjellstrom et al. 1989). This finding further supports our use of a loss of 1.5 IQ points for each doubling in our base case analysis. Confounders such as polychlorinated biphenyls did not cause significant confounding of the data in the Faroe Islands study (Budtz-Jorgensen et al. 2002; Steuerwald et al. 2000). As a conservative measure, we nonetheless chose to set as outer bounds for the impact on intelligence of methyl mercury exposure a range of IQ decrements from 0.85 to 2.4 IQ points per doubling, as described by the Faroes researchers (Jorgensen et al. 2004). In applying the EAF methodology, we assume that the relationship between cord blood mercury and IQ is relatively linear over the range of exposures studied (> 5.8 μg/L).

In our sensitivity analysis, we used the same linear dose–response model that was selected by the National Research Council in setting a reference dose for mercury exposure (National Research Council 2000). The Faroes researchers found that, for those children whose mothers had hair mercury concentrations < 10 μg/g, a 1-μg/L increase of cord blood mercury concentration was associated with adverse impacts on neurodevelopmental tests ranging from 3.95 to 8.33% of a standard deviation, or 0.59–1.24 IQ points (average = 0.93 IQ points) (Jorgensen et al. 2004). We also varied the cord:maternal blood mercury ratio from 1 to 1.7 in calculating IQ impact from the linear model as part of our sensitivity analysis. As an upper bound to our cost estimate using the logarithmic model, we calculated the economic cost assuming that children born to women with mercury concentrations 3.5–4.84 μg/L suffer no loss in cognition and that successive portions of the birth cohort experience losses of cognition of 1.21, 1.84, 2.55, and 5.13 IQ points, respectively. The lower-bound estimate assumed that children born to women with mercury concentrations 4.84–5.8 μg/L suffer no loss in cognition and that successive portions of the birth cohort experience losses of cognition of 0.22, 0.48, and 1.39 IQ points.

As an upper bound to our cost estimate using the linear model, we calculated the economic cost assuming that children born to women with mercury concentrations 3.5–4.84 μg/L suffer no loss in cognition and that successive portions of the birth cohort experience losses of cognition of 3.01, 5.04, 7.84, and 24.43 IQ points, respectively. The lower-bound estimate assumed that children born to women with mercury concentrations 4.84–5.8 μg/L suffer no loss in cognition and that successive portions of the birth cohort experience losses of cognition of 0.56, 1.35, and 5.99 IQ points.

### Calculation of economic costs of IQ loss.

To estimate the costs associated with the cognitive and behavioral consequences of mercury exposure, we relied on an economic forecasting model developed by Schwartz et al. (1985), and we applied this model to NHANES data on prevalence of mercury exposure in women of childbearing age (Schober et al. 2003; Schwartz et al. 1985). In this model, lead concentrations are assumed on the basis of work by Salkever (1995) to produce a dose-related decrement in IQ score. Those decrements in IQ are, in turn, associated with lower wages and diminished lifetime earning power. Salkever used three regression techniques to derive direct and indirect relationships among IQ, schooling, probability of workforce participation, and earnings. He estimated a percentage in lost earnings per IQ point from the percent loss of earnings for each microgram per deciliter increase in blood lead level. Salkever found a 0.473 point decrement in lost lifetime earnings for each microgram per deciliter increase among men and a 0.806 point decrement for each microgram per deciliter increase among women (Salkever 1995). Using Schwartz’s (1994) estimate that 0.245 IQ points are lost for each microgram per deciliter increase in blood lead, Salkever (1995) estimated a percentage loss in lifetime earnings per IQ point among men (1.931%) and women (3.225%). We assume that this relationship remains linear across the population range of IQ.

Assuming an annual growth in productivity of 1% and applying a 3% real discount rate, the present value of lifetime expected earnings is $1,032,002 for a boy born in 2000 and $763,468 for a girl born in the same year (Max et al. 2002). The costs of the diminution in this earning power were calculated for the 2000 American birth cohort, using available data on the number of male and female births in 2000 [Centers for Disease Control and Prevention (CDC) 2002a]. We diminished our cost estimate by 0.69%, the infant mortality rate in 2000, to account for those children for whom methyl mercury exposure is unlikely to result in diminished economic productivity (CDC 2002b).

### American sources of mercury emission.

Mercury emissions result from anthropogenic as well as from natural sources, and we limited our analysis to methyl mercury derived from anthropogenic sources. The UNEP recently estimated that anthropogenic uses account for 70% of the 5,500 tons of mercury released into the earth’s atmosphere worldwide (UNEP 2002). Therefore, to limit our analysis to anthropogenic mercury, we applied a 70% factor to convert the cost of lost economic productivity resulting from methyl mercury exposure to the cost attributable to anthropogenic methyl mercury exposure.

We next parsed out the proportion of anthropogenic methyl mercury in fish that arises from American sources and then isolated the subset of that proportion that is emitted by coal-fired electrical generating plants. In 1995, the most recent year for which federal data on the relative deposition of mercury from American and other global sources are available, 158 tons of mercury were emitted to the atmosphere by American anthropogenic sources. Fifty-two (33%) of those 158 tons were deposited in the lower 48 states, whereas the remaining two-thirds were added to the global reservoir (U.S. EPA 2004d). Also in 1995, an additional 35 tons of mercury from the global reservoir were deposited in the United States. Therefore, a total of 87 total tons of mercury were deposited in the United States in that year, of which 60% (52 of 87) were attributable to American anthropogenic sources (U.S. EPA 1996, 1997). This mercury would have been available to bioaccumulate in the marine and aquatic food chains and to enter American freshwater and saltwater fish.

Further complicating our calculations is the fact that not all of the fish sold in America is from American sources. Of the 10.4 billion pounds of edible fish supplied in the United States in 2002, 4.4 billion (42%) are imported from sources outside of the United States (National Marine Fisheries Service 2002). Because U.S. emissions account for 3% of global emissions (UNEP 2002; U.S. EPA 1996), we calculate that the mercury content of imported fish is 2% of American anthropogenic origin: 158 tons of American emissions – 52 tons of American mercury deposited on American soil = 106 tons of American mercury available to contaminate imported fish; 5,500 tons emitted globally – 87 tons deposited on American soil = 5,413 tons of mercury from all sources to contaminate imported fish; 106 tons of mercury available/5,413 tons of mercury from all sources = 0.02, or 2% of mercury in imported fish of American origin. In the remaining 58% of fish consumed in the United States, we assume that 60% of the mercury content comes from American anthropogenic sources (U.S. EPA 1996, 1997). We therefore applied a 36% factor (the weighted average of American sources of mercury content in fish, or 0.6 × 0.58 + 0.02 × 0.42) to specify the economic costs of anthropogenic methyl mercury exposure attributable to American sources.

Modeling supported by the Electric Power Resource Institute (EPRI) estimates that 70% of the mercury deposited in the United States comes from foreign sources (Seigneur et al. 2004). This EPRI analysis also finds that U.S. sources are responsible for > 60% of mercury deposition in the Boston–Washington, D.C. corridor. In one of the model’s selected receptor areas—Pines Lake, New Jersey—80% of the deposition originated from U.S. sources, showing that regional deposition can be higher than the 60% number we use in this analysis (Seigneur et al. 2004). In our sensitivity analysis, we varied the factor used to convert the economic cost of anthropogenic methyl mercury exposure to the economic cost attributable to American sources from 18% (0.3 × 0.58 + 0.02 × 0.42, using EPRI’s modeling) to 36% (using federal data on mercury deposition) (Seigneur et al. 2004).

In 1999, the most recent year for which data on American mercury emissions are available, 48 (41%) of the 117 tons of mercury emissions from anthropogenic sources in the United States were emitted by electric power generation facilities (U.S. EPA 2003a). To calculate the economic cost of methyl mercury exposure attributable to these facilities, we applied an additional fraction of 41% in our analysis.

## Results

### Base-case analysis.

Each year in the United States, between 316,588 (7.8% of the annual birth cohort) and 637,233 babies are born with cord blood mercury levels > 5.8 μg/L. The lower-bound estimate of 316,588 babies is based on the very conservative assumption that maternal and cord blood mercury concentrations are equal. But if the cord blood mercury concentration is on average 70% higher than the maternal blood mercury concentration, as suggested by recent research (Stern and Smith 2003), 637,233 babies, or 15.7% of the birth cohort, experience cord blood mercury levels > 5.8 μg/L. Fetal blood mercury levels > 5.8 μg/L are associated with small but significant loss of IQ. This decrement in IQ appears to be permanent and irreversible, and it adversely affects a significant portion of the annual birth cohort’s economic productivity over a lifetime.

Using our base-case assumptions (impact for women with total mercury > 4.84 μg/L, cord:maternal mercury ratio = 1.7, IQ impact = 1.5 points per doubling), we calculated costs for the 405,881 children who suffer IQ decrements resulting from fetal methyl mercury exposure. Under these assumptions, 89,293 children suffered a 0.76 decrement in IQ and another 113,647 experienced a 1.15 IQ point decrement. The 5% most highly exposed children in the 2000 birth cohort suffered subclinical losses in IQ in our model ranging from 1.60 to 3.21 points. Although this diminution in intelligence is small in comparison with the loss of cognition that can result from other genetic and environmental processes, the loss resulting from methyl mercury exposure produces a significant reduction in economic productivity over a lifetime. We estimate the aggregate cost of the loss in IQ that results from exposure of American children to methyl mercury of anthropogenic origin to be $8.7 billion (all costs in 2000 US$) annually (Table 1).

### Sensitivity analysis.

We estimate that the cost of anthropogenic methyl mercury exposure ranges from $2.2 billion (impact only for the 316,588 children born to women with total mercury > 5.8 μg/L, IQ impact = 0.85 points per doubling) to $13.9 billion (impact for the 405,881 women with total mercury > 4.84 μg/L, IQ impact = 2.4 points per doubling). Using the linear dose–response model that was selected by the National Research Council in recommending a reference dose for mercury exposure (a model that predicts an average loss of 0.93 IQ points per 1-μg/L increase in mercury concentration) (Jorgensen et al. 2004; National Research Council 2000), we find that the environmentally attributable cost of methyl mercury exposure is $32.9 billion, assuming a cord:maternal blood mercury ratio of 1.7. Employing a linear model and assuming that the true loss in IQ resulting from a 1-μg/L increase in blood mercury ranges from 0.59 to 1.24 points, we find that the outer bounds of our estimate range from $7.0 billion (impact only for women with total mercury > 5.8 μg/L, IQ impact = 0.59 points per μg/L increase, cord:maternal mercury ratio = 1) to $43.8 billion (impact for women with total mercury > 4.84 μg/L, IQ impact = 1.24 points for each microgram per deciliter increase, cord:maternal mercury ratio = 1.7) (Table 2).

### Sources of costs.

After applying the 36% fraction to restrict our analysis to American anthropogenic sources, we estimate that the attributable cost of methyl mercury exposure to the developing fetus from American anthropogenic sources is $3.1 billion annually, using the logarithmic model developed by the Faroes researchers (Grandjean et al. 1999; Jorgensen et al. 2004) and assuming a 1.5-point IQ impact for each doubling of methyl mercury exposure (Budtz-Jorgensen et al. 2002). Our sensitivity analysis, in which we also varied the attributable fraction for American sources from 18% (industry data sources) to 36% (federal data sources) (Seigneur et al. 2004; U.S. EPA 1996, 1997), suggests that the true cost of methyl mercury exposure from American emissions ranges from $0.4 to $15.8 billion annually.

**Figure 1:**
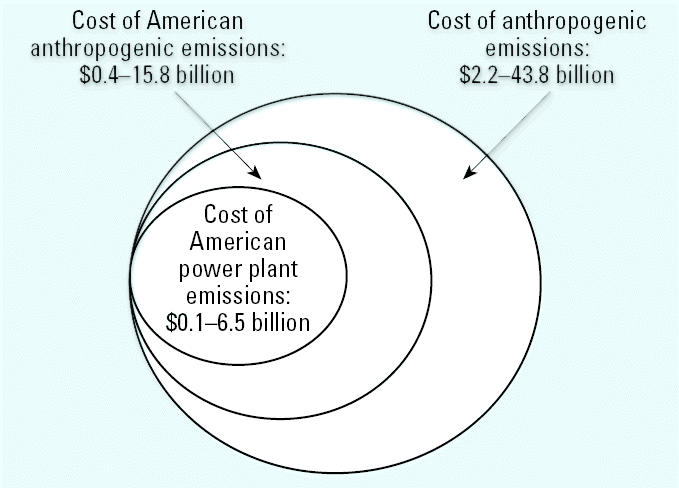
Portions of cost of methyl mercury exposure attributed to sources. Assumptions: 18–36% attributable to American sources; 41% of American emissions attributable to American power plants.

To focus specifically on the costs of fetal exposure to mercury released by American coal-fired power plants, we examined the impact of the 41% of U.S. anthropogenic emissions of mercury attributable to these facilities. We estimate that the attributable cost of methyl mercury exposure from American electric generation facilities to the developing fetus is $1.3 billion. Applying our sensitivity analysis in this model, we find that the true cost of methyl mercury exposure from electric generation facilities to the American birth cohort ranges from $0.1 to $6.5 billion/year (Figure 1). Again, the major source of these costs is loss of earnings over a lifetime.

## Discussion

The major findings in this analysis are *a*) that exposure to methyl mercury emitted to the atmosphere by American electric generation facilities causes lifelong loss of intelligence in hundreds of thousands of American babies born each year and *b*) that this loss of intelligence exacts a significant economic cost to American society, a cost that amounts to at least hundreds of millions of dollars each year. Moreover, these costs will recur each year with each new birth cohort as long as mercury emissions are not controlled. By contrast, the cost of installing stack filters to control atmospheric mercury emissions is a one-time expense. The high costs of *in utero* exposure to methyl mercury are due principally to the lifelong consequences of irreversible injury to the developing brain. Similar lifelong neurobehavioral consequences have been observed after exposure of the developing brain to other environmental toxicants, including lead (Baghurst et al. 1987; Bellinger 2004; Dietrich et al. 1987; Opler et al. 2004; Wasserman et al. 2000), polychlorinated biphenyls (Jacobson and Jacobson 1996), and ethanol (Lupton et al. 2004).

Because the literature has presented a range of possible consequences for methyl mercury toxicity, we have provided a range of possible public health and economic consequences. This range is meant to inform the choices that environmental and public health officials make in protecting vulnerable populations from methyl mercury exposure. Our range for the true economic costs of methyl mercury toxicity to the developing brain omits the cost of exposures to the 231,352 children born to women in 2000 with blood mercury concentrations between 3.5 and 4.84 μg/L. If the true cord blood ratio is 1.7 times the maternal blood concentration, as described in the most recent and extensive meta-analysis on the matter (Stern and Smith 2003), these children are also born with cord blood mercury concentrations above the 5.8 μg/L concentration at which adverse neurodevelopmental impact has been found. We chose not to include them in our analysis because other studies have found lower ratios and because we restricted ourselves in this analysis to the use of available, published prevalence data of maternal blood mercury concentrations. In our sensitivity analysis, we also selected low cord:maternal blood ratios so as to describe most accurately the range of values for the true cost of methyl mercury exposure to the developing fetus.

Our analysis also omits the cost of the cardiovascular impacts of mercury exposure (Grandjean et al. 2004) or the costs of mercury exposure to children in the first 2 years of postnatal life, when myelination is still continuing and the blood–brain barrier remains vulnerable to penetration by methyl mercury (Rodier 1995). We chose not to include these aspects of methyl mercury toxicity in our range of estimates at this time because there do not exist sufficient quantitative data to permit construction of a reliable model.

A limitation on our analysis is that it did not consider other societal costs beyond decreased lifetime earnings that may result from exposure of the developing brain to methyl mercury. For example, if the value of a child’s social productivity is approximately $4–9 million, as suggested by studies of willingness-to-pay (WTP) estimates of a life (Viscusi and Aldy 2004), then by the WTP methodology the true cost of methyl mercury toxicity may be much higher than our estimate. We also chose not to include other noncognitive impacts. Lead, for example, has been associated with criminality and antisocial behavior (Dietrich et al. 2001; Needleman et al. 1996, 2002; Nevin 2000; Stretesky and Lynch 2001). However, because these behaviors have not been described as yet for methyl mercury, we chose not to include such costs in our estimate.

Some will argue that our range of costs fails to incorporate the role of confounding factors in quantifying the economic consequences of methyl mercury exposure. It is true that efforts to delineate the potential synergistic role of methyl mercury and other chemicals in mediating neurocognitive and other effects are bedeviled by lack of knowledge about possible interactions and synergies among chemicals or between chemicals and other environmental hazards, even though the environment of a child includes mixtures of chemical and biologic toxicants. Only a study of the magnitude of the National Children’s Study will facilitate simultaneous examination of the effects of multiple chemical exposures, of interactions among them, and of interactions among biologic, chemical, behavioral, and social factors (Trasande and Landrigan 2004). However, we note that loss of cognition resulting from methyl mercury exposure in the Faroe Islands study remained evident when blood levels of polychlorinated biphenyls, which are known fetal neurotoxicants (Jacobson and Jacobson 1996), were included in the analysis (Budtz-Jorgensen et al. 2002; Steuerwald et al. 2000).

We note the U.S. EPA’s recent success in minimizing mercury emissions from medical waste (U.S. EPA 2004e) and municipal incinerators (U.S. EPA 2004f, 2004g), actions that resulted in a decrease in total mercury emissions by at least 80 tons per year from 1990 to 1999 (U.S. EPA 2003b). Although data are not available on blood mercury concentrations over the past decade that followed from those actions, the impact of these reductions is likely to have been substantial.

Some commentators have used data from the Seychelles study to argue that methyl mercury is not toxic to the fetus at low concentrations and to suggest that fear of mercury exposure is needlessly preventing women from ingesting fish and thus denying them access to beneficial long-chain polyunsaturated fatty acids (LCPUFAs), especially docosahexaenoic acid (DHA). We do not dispute that DHA and other LCPUFAs are important for optimal development of the fetal visual and nervous systems (Innis 1991). The human fetus has a limited ability to synthesize DHA’s precursor, α-linolenic acid, and therefore it must be largely supplied from maternal sources (Carnielli et al. 1996; Larque et al. 2002; Szitanyi et al. 1999). We also note a report that associated an average monthly decline in fish consumption of 1.4 servings among Massachusetts women with a U.S. Food and Drug Administration advisory on the health risks of mercury (Oken et al. 2003). Nonetheless, the American Heart Association, a strong advocate for the cardio-protective effects of LCPUFAs, recommends that children and pregnant and lactating women avoid potentially contaminated fish (Kris-Etherton et al. 2002). Fish advisories should not recommend that consumers abstain from fish, but they should assist in choosing the best kinds of fish to eat. Lists of fish that are safe and unsafe from the perspective of mercury exposure have been published and made widely available to consumers (U.S. EPA 2004h).

Early reports of disease and dysfunction of environmental origin in children have on repeated occasions failed to produce proactive response to protect children. The long history of lead use in the United States provides a chilling reminder of the consequences of failure to act on early evidence of harm. It is important that we not repeat this sequence with mercury. Within the last century, as a result of increased industrial activity, mercury emissions worldwide have increased 2- to 5-fold, and anthropogenic emissions now surpass emissions from natural sources (Nriagu 1989).

The data from this analysis reinforce the results of recent epidemiologic studies and indicate an urgent need on economic grounds for regulatory intervention at the federal level to minimize mercury emissions. Our analysis captures the cost of methyl mercury exposure for only 1 year’s birth cohort, but the cost of mercury exposure will continue to accrue in each succeeding year if power plants fail to install flue gas filters (U.S. Department of Energy 2004) or to implement other technologies to reduce mercury emissions. The cost savings from reducing mercury exposure now will provide savings in improved productivity and enhanced national security for generations to come.

## Figures and Tables

**Table 1:** Cost of anthropogenic mercury (Hg) exposure using a logarithmic model.

	Segment of population (percentile)			
Variable	90–92.1 Hg	92.2–94.9 Hg	95–99.3 Hg	≥99.4 Hg
Range of maternal total Hg concentration	4.84–5.8 μg/L	5.8–7.13 μg/L	7.13–15.0 μg/L	> 15.0 μg/L
Assumed maternal total Hg concentration	4.84	5.8	7.13	15
No effect concentration (maternal total Hg)	3.41	3.41	3.41	3.41
IQ points lost at assumed concentration	0.76	1.15	1.60	3.21
Loss of 1 IQ points = decrease in lifetime earnings				
For boys, lifetime earnings (1.931% decrease)	$1,032,002				
For girls, lifetime earnings (3.225% decrease)	$763,468				
No. of boys in birth cohort affected	45,693	58,155	91,387	12,462
No. of girls in birth cohort affected	43,601	55,492	87,201	11,891
Lost income	$1.1 billion	$2.0 billion	$4.4 billion	$1.2 billion
Total cost = $8.7 billion in each year’s birth cohort				

Assumptions: EAF = 70%, main consequence = loss of IQ over lifetime.

**Table 2:** Sensitivity analysis: cost of anthropogenic methyl mercury exposure.

Variable	Base-case cost estimate (range)*^a^*
Children born to women with Hg > 4.84 μg/L, effect > 3.5 μg/L	
Logarithmic model	$8.7 billion ($4.9–13.9 billion)
Linear model, cord:maternal Hg ratio = 1.7	$32.9 billion ($20.9–43.8 billion)
Linear model, cord:maternal Hg ratio = 1	$19.3 billion ($12.3–25.8 billion)
Children born to women with > 5.8 μg/L, effect > 4.84 μg/L	
Logarithmic model	$3.9 billion ($2.2–6.3 billion)
Linear model, cord:maternal Hg ratio = 1.7	$18.7 billion ($11.9–24.9 billion)
Linear model, cord:maternal Hg ratio = 1	$11.0 billion ($7.0–14.6 billion)
Range of estimates	
Logarithmic model	$2.2–13.9 billion
Linear model	$7.0–43.8 billion

Assumptions: EAF = 70%, main consequence = loss of IQ over lifetime.

a Based on range of possible IQ decrement:increase cord blood mercury.
